# Motion Analysis of Match Play in U14 Male Soccer Players and the Influence of Position, Competitive Level and Contextual Variables

**DOI:** 10.3390/ijerph18147287

**Published:** 2021-07-07

**Authors:** Erling Algroy, Halvard Grendstad, Amund Riiser, Tone Nybakken, Atle Hole Saeterbakken, Vidar Andersen, Hilde Stokvold Gundersen

**Affiliations:** 1 Campus Bergen, NLA University College, 5812 Bergen, Norway; 2 Department of Sport, Food and Natural Sciences, Campus Bergen, Western Norway University of Applied Sciences, 5020 Bergen, Norway; halvardg@nih.no (H.G.); tone.nybakken@hvl.no (T.N.); hilde.stokvold.gundersen@hvl.no (H.S.G.); 3 Department of Sport, Food and Natural Sciences, Campus Sogndal, Western Norway University of Applied Sciences, 6851 Sogndal, Norway; amund.riiser@hvl.no (A.R.); atle.saeterbakken@hvl.no (A.H.S.); vidar.andersen@hvl.no (V.A.)

**Keywords:** match running performance, youth soccer, positional differences, competitive levels, contextual variables

## Abstract

This study aimed to investigate match running performance in U14 male soccer players in Norway, and the influence of position, competitive level and contextual factors on running performance. Locomotion was monitored in 64 different U14 players during 23 official matches. Matches were played at two different competitive levels: U14 elite level (*n* = 7) and U14 sub-elite level (*n* = 16). The inclusion criterion was completed match halves played in the same playing position. The variables’ influence on match running performance was assessed using mixed-effect models, pairwise comparisons with Bonferroni correction, and effect size. The results showed that the U14 players, on average, moved 7645 ± 840 m during a match, of which 1730 ± 681 m (22.6%) included high-intensity running (HIR, 13.5–18.5 km·h^−1^) and sprinting (>18.5 km·h^−1^). Wide midfielders (WM) and fullbacks (FB) covered the greatest sprint distance (569 ± 40 m) and, in addition to the centre midfield position (CM), also covered the greatest total distance (TD) (8014 ± 140 m) and HIR distance (1446 ± 64 m). Centre forwards (CF) performed significantly more accelerations (49.5 ± 3.8) compared other positions. TD (7952 ± 120 m vs. 7590 ± 94 m) and HIR (1432 ± 57 m vs. 1236 ± 43 m) were greater in U14 elite-level matches compared with sub-elite matches. Greater TD and sprint distances were performed in home matches, but, on the other hand, more accelerations and decelerations were performed in matches played away or in neutral locations. Significantly higher TD, HIR and sprinting distances were also found in lost or drawn matches. In conclusion, physical performance during matches is highly related to playing position, and wide positions seem to be the most physically demanding. Further, competitive level and contextual match variables are associated with players’ running performance.

## 1. Introduction

In the last decade, several studies have described the physical demands of youth soccer [1]. Previous reports have shown that U14 players cover a total distance between 105–115 m·min^−1^ during a match and 11.5–14.5 m·min^−1^ at higher speed (13.1–19 km·h^−1^) [2,3] Additionally, elite U14 players perform about 0.4 ± 0.2 sprints (speed above 25.2 km·h^−1^) [2] and 1.82 ± 0.33 accelerations per min during a match [2,4]. Although there is a growing body of evidence related to the physical match characteristics of youth soccer players, very few studies have examined running performance at the U14 level specifically in relation to high-explosive actions like sprints, accelerations and decelerations [1,5]. Due to the increasing physical match demands with age, more knowledge about performance at specific age levels is necessary to gain insights into the prerequisites of competing at U14 level. Additionally, the influence of competing standard and positional roles on players’ running performance seems to be an important factor in youth soccer [1] and must be accounted for.

Although the scientific evidence is scarce, some studies have examined match running characteristics among youth players. These have reported that U13–U18 players’ physical match characteristics are affected by positional demands, as strikers and wide midfielders demonstrated the highest peak game speeds and frequency of high-intensity actions [5,6]. Additionally, centre midfielders have been reported to cover the highest total distance [5,6], whereas centre backs covered the lowest total distance [7] and the lowest amount of high-intensity actions [6].

A current limitation in research on youth soccer is the lack of information regarding the influence of contextual variables like match results, location and match status [1]. One previous study has shown that U14 elite level players outperform non-elite players regarding match running performance [2], suggesting a higher external demand during elite matches [7,8]. Studies from senior soccer have also reported greater match running performance in games lost compared with games won [9].

To date, no study has examined the match performance characteristics of U14 soccer players from Norway or other Scandinavian countries. Comparing data from different countries and regions of the world would be important to enhance our understanding of various approaches to and philosophies of talent development. Especially, more research regarding accelerations and decelerations is necessary in youth soccer. These high-intensity actions have been found to be crucial determinants for successful performance and to discriminate high- and low-level adult players [10]. Accelerations and decelerations also require high rates of force development and are therefore related to the total match load [11]. Additionally, studies on U14 players have, to the best of our knowledge, only been conducted in the United Kingdom, Qatar and New Zealand [1]. Hence, the first aim of this study was therefore to analyse match running performance in U14 male soccer players in Norway. A second aim was to identify how playing position, competitive level and contextual factors influenced running performance. We hypothesise that players’ playing positions and competitive level influenced the amount of high-intensity actions in U14 soccer matches.

## 2. Materials and Methods

The current study is part of a longitudinal research project, examining factors related to talent development in youth soccer [12]. The study design in the present study is descriptive, and match running performance data were collected during the 2018 season, from April to October. Collection of anthropometric data was performed during 2 different days for each player in the middle of the 2018 season. Contextual factors investigated in the present study were match results, match location and match halves.

### 2.1. Participants

Sixty-four U14 male outfield players (age: 14.0 ± 0.3 years, height: 166.3 ± 8.8 cm, weight: 51.9 ± 9.7 kg, body fat: 8.7 ± 3.3%) from 4 clubs with youth soccer academies were included. Three of the players were U13 players but were included as they played in the U14 teams investigated. The Regional Committee for Medical and Health Research Ethics approved the study (2017/1731), which was conducted in accordance with the Helsinki Declaration. Since players were under the legal age of consent, both the players and their parents gave written informed consent to participate. All results were treated anonymously.

### 2.2. Matches

Match running performance was obtained from 23 matches, 15 at the team’s home location and 5 away and 3 on a neutral field. In 17 of the matches, the teams won; 1 match ended with a draw and 5 with a loss. All matches were official league or cup matches, played outdoor on regular-sized synthetic grass soccer fields with 11 players per side. Matches were played at 2 different competitive levels: U14 elite, national level (7 matches, 93 match halves), U14 sub-elite, the highest regional level (16 matches, 249 match halves). Players playing in elite teams are recruited into playing at professional youth academies and matches are played in the U14 national level league, in which youth academies from professional Norwegian clubs participate. Some players played matches at more than 1 level.

Match playing time was 2 × 35 min in the U14 local series and 2 × 40 min for the U14 national series. All match performance outcomes were normalised to 35 min of playing time before the analyses to allow direct comparison between matches at different competing levels and to be comparable with other studies examining U14 players.

Because of the “rolling substitution” policy resulted in a large variation in playing time between players, all match halves completed by 1 player in the same playing position were included in the dataset. The dataset comprised 342 match halves, including 187 first halves and 155 second halves; 278 of the included halves were from complete matches played by 1 player.

### 2.3. GPS Tracking

During the matches, the players wore a portable and previously validated [13] GPS device (Apex, STATSsport, Newry, UK) monitoring their motions and position with a sampling frequency of 18 Hz. The players wore, in most cases, the same unit in every game to limit inter-unit reliability issues, although the present GPS system has been shown to have excellent inter-unit reliability [14]. The units were placed in vests located between the players’ scapulae. The raw GPS data were synchronised to the start and end of each half match period and exported for further analysis.

### 2.4. Match Running Categories

Match activities were divided into different speed categories: walking (0.1–4.5 km·h^−1^), low-intensity running (LIR, 4.5–8.5 km·h^−1^), medium-intensity running (MIR, 8.6–13.5 km·h^−1^), high-intensity running (HIR, 13.6–18.5 km·h^−1^), sprinting (>18.5 km·h^−1^) and maximal speed. The speed thresholds were age-specific [3] and adapted from previous studies on U14 soccer players [3,15] to compare running performance with previously reports on U14 soccer players. In addition, sprint distance with a threshold adapted from studies on senior soccer players (25.2 km·h^−1^ with a duration of at least 1 s) was included to compare sprint distance between U14 players and senior elite players [16]. Total distance included all locomotion during the match. Data are presented both in absolute (m) and relative (m·min^−1^) distances.

The number of accelerations and decelerations was also examined, and were defined as actions when speed was increased or decreased by more than 3 m·s^−2^ and lasted more than 0.5 s. Only accelerations and decelerations at speeds above 13.5 km·h^−1^ were included.

### 2.5. Playing Position

Players were categorised as centre backs (CB, *n* = 70 match halves), fullbacks (FB, *n* = 68 match halves), centre midfielders (CM, *n* = 100 match halves), wide midfielders (WM, *n* = 65 match halves) or centre forwards (CF, *n* = 39 match halves). Players swapping positions were excluded from the analysis. Goalkeepers were excluded from the study due to their low running demands [16,17].

### 2.6. Statistical Analyses

Data are presented as means with the standard deviation (SD) or 95% confidence interval (CI). Visual inspection confirmed that all data were normally distributed. Differences in match running performance between halves were assessed by one-way ANOVA analysis.

The fixed effects (E) of the independent variables on match running performance were assessed using mixed-effect models with the player as a random effect to adjust for multiple match observations by the same player. All independent variables were included in the models, as they may influence match running performance in theory. The variables analysed were playing position (5 positions), competitive level (elite vs. sub-elite), match location (home vs. neutral/away), match results (win vs. draw/loss) and match half (first vs. second half). The collinearity between the continuous independent variables was inspected by Pearson’s product moment correlations coefficients and were included if *r* < 0.6. Pairwise comparisons for competition level and playing position were performed with Bonferroni correction. IBM SPSS Statistics (version 26, IBM, Armonk, NY, USA) was used for all statistical analyses. Significance for all analyses was accepted at *p* ≤ 0.05. When significant differences were detected, Cohen’s *d* effect size (ES) was calculated. An ES of 0–0.2 was considered trivial, >0.2 as small, >0.5 as medium and >0.8 as large [18].

## 3. Results

### 3.1. Match Running Performance

During a match, players covered, on average, 22% of the total distance by high-intensity running (13.6–18.5 km·h^−1^) and sprinting (>18.5 km·h^−1^), 32% by medium-intensity running (8.6–13.5 km·h^−1^), 32% by low-intensity running (4.6–8.5 km·h^−1^) and 16% by walking (0.1–4.5 km·h^−1^). More distance was covered in the first compared with the second half, both in metres (ES = 0.71) and metres pr minute (ES = 0.29). Total distance and high-intensity activities for each half are presented in Table 1.

### 3.2. Playing Position

Playing positions were associated with TD and all high-intensity parameters investigated (HIR, sprints, accelerations and decelerations). FB, WM and CM showed significantly greater total distance and HIR distance compared with CB (ES_TD_ = 0.62–1.05 and ES_HIR_ = 0.91–3.06) and CF (ES_TD_ = 0.62–1.05 and ES_HIR_ = 0.57–2.61). Wide playing positions (WM, FB) covered the most sprint distance (ES = 0.64–1.08), and CF also covered greater sprint distance compared with CB positions (ES = 0.90). CF performed significantly more accelerations compared with all other playing positions (ES = 0.48–0.96). WM and CF had the highest number of decelerations (ES = 0.38–1.04), and CB performed significantly fewer decelerations compared with all other positions (ES = 0.53–1.18). Positional differences according to running performance are described in Table 2.

### 3.3. Competitive Level

Players covered significantly greater total distance (ES = 0.35) and HIR distance (ES = 0.43) (13.6–18.5 km·h^−1^) in matches played at U14 elite level compared with the local U14 sub-elite level (Table 3). No differences between groups were found regarding sprints, accelerations and decelerations.

### 3.4. Contextual Factors

Significant greater total distances (*p* < 0.001, ES = 0.36), HIR distances (*p* < 0.001, ES = 0.32) and sprint distances (*p* = 0.016, ES = 0.11) were observed in matches lost or drawn compared with matches won. Match location was also shown to have an impact on most running variables investigated. Players performed greater TD (*p* = 0.043, ES = 0.13) and sprint distance (*p* = 0.038, ES = 0.20) when playing at home compared with playing away or at a neutral match location. On the other hand, the number of accelerations (*p* < 0.001, ES = 0.56) and decelerations (*p* < 0.001, ES = 0.46) was higher when playing away or at a neutral location compared with playing at home. Greater total distances (3883 ± 405 vs. 3762 ± 435, ES = 0.71) covered in one half were observed (*p* = 0.008), but we found no other differences between match halves.

## 4. Discussion

This study aimed to analyse match running performance in U14 male soccer players in Norway, and to identify how playing position, competitive level and contextual match variables influenced running performance. Overall, 22% of the locomotion during matches constituted high-speed running and sprinting, with the rest being performed at lower running intensities. There were significant differences in physical performance between positions, in addition to greater running demands when players competed at the highest competitive standard. Furthermore, contextual factors were associated with most running variables investigated.

The findings of the present study showed that Norwegian male U14 soccer players run similar distances or more during a match compared with previous reports on U14 players [1]. The group in our study covered a total distance of 109.2 m·min^−1^, which is higher than that of 95.7 m·min^−1^ reported in New Zealand U14 male soccer players [3] and 106.5 m·min^−1^ in English U14 soccer players [19]. Similarly, in our study, 22% of the total match time was spent as high-intensity running actions (>13.6 km/h^−1^), whereas reports from youth soccer in New Zealand [3], Italy [20] and England [19] have shown total high-intensity running actions to constitute 7%, 16% and 17%, respectively, with a similar speed threshold applied as in the present study. These results could be explained by how the different countries approach and emphasise the game, as studies from senior soccer highlighted that soccer philosophy, technical performance and physical performance varied between different countries and leagues [21,22,23].

Across playing positions we found that CF had a greater number of accelerations compared with all other playing positions. In contrast, a previous study on youth soccer showed that attackers perform a high amount of accelerations, but that players in wide positions (WM and FB) in general perform more accelerations than central players (CB, CM and CF) [24]. However, Vigh-Larsen and colleagues [24] only examined 14 outfield players from one single team, and their findings could reflect the team’s formation and tactical dispositions, or the physical capacity of the individual players [25]. Indeed, discrepancies have been found for high-intensity physical performance between different playing formations in elite senior soccer [26]. Regardless, our finding that CB perform fewer accelerations than other positions is in line with previous findings [24,27], and also that CB positions in general have a lower running performance compared with other playing positions [1]. Our results showed that wide playing positions performed the highest amount of high-intensity work and, in general, were the most physically demanding playing positions. This was also supported by Pettersen and colleagues (2019), who investigated a group of Norwegian U17 soccer players [27]. In senior soccer, studies have demonstrated that evolving tactics over the last decade have especially impacted the physical demands of wide players, as this position has shown the greatest increase in high-intensity running [17,28]. Our findings suggest that the high running demands of wide positions found at higher age groups and in senior soccer also seems to be evident at U14 level.

Total distance and high-intensity running were significantly higher in matches at U14 elite level compared with sub-elite level, which is in line with findings from a previous study [2]. However, the observed difference was small regarding effect size, and we found no differences regarding sprint distance, or the number of accelerations and decelerations. A possible explanation is that the sub-elite teams represented in our study were the highest ranked sub-elite teams and included highly talented players also recruited in the U14 national and regional teams. The observed difference in our study regarding running performance and competitive level might been greater with a wider range of teams included from the sub-elite group. Our finding is in contrast to what has previously been reported in top-ranked U17 players, where the best teams outperformed players in lower-ranked teams [29]. Contrarily, studies on senior players from teams that finished in the top five at the end of the English Premier League season performed less sprinting than those that finished outside of the top five [30].

Match results were associated with running performance. The players covered a greater total distance, HIR distance and sprint distance in matches they lost or drew compared with the matches they won. Previous studies in elite senior soccer have shown conflicting results regarding the influence of match results, where Castellano et al. [9] found no relationship with match results, whereas Lago et al. [31] found that players ran less at higher intensities when winning. Playing against high-quality opposition has been found to be associated with lower ball possession [32], and a possible explanation could be that the lower-quality team has to cover a greater distance to regain possession and defend important spaces. Our results also showed that match location influenced match running performance, as greater TD and sprinting distance (>18.5 km·h^−1^) were performed when playing at home, even though fewer accelerations and decelerations were performed. Different tactical approaches to home matches versus away matches may be an explanation, but more research is necessary to better understand this. A limitation in the present study according to contextual factors is the preponderance of matches ending with a win compared with matches ending with a draw/loss. Our results showed a significant association between contextual factors and several running performance variables; however, these findings had, in general, a small/medium effect size. More research is necessary in order to better understand the link between match running performance and contextual factors in youth soccer. The influence of these multivariate factors should not be considered in isolation.

A challenge when comparing time motion data from different studies from youth soccer is the different methodological approach to the rolling substitution policy, resulting in a large variety in playing time between players. In the present study, all completed match halves were included to increase the sample size and statistical power, in contrast to most previous studies that only included completed matches. Excluding players who did not play full matches may bias analyses by reducing the sample size and removing variations in running performance. This is important to take into account when interpreting positional differences, as offensive positions are commonly substituted [33]. We observed a significantly lower relative total distance covered in the second half (3.9 m·min^−1^), but the small difference observed between halves was argued to be of less practical meaning.

Overall, our results show that physical performance during matches is affected by several factors and differs between leagues and countries. Running demands in soccer and especially different positional running demands are evolving [28], and highlight the need for updated research. This is the first study to assess match-related physical performance and positional differences in Scandinavian U14 youth soccer. Our results suggest that physical performance during matches is similar or higher than in reports from other parts of the world and are highly related to playing position. Wide playing positions seem to be the most physically demanding positions. In addition, centre forwards perform more accelerations than all other positions. Given the high physical demands of high-intensity work and the impact of these actions on post-match muscle damage [34], coaches must consider different positional match loads, and individualise training according to positional demands. Further, our data indicate that playing at the highest U14 level was more physically demanding, suggesting a higher external demand during elite matches compared with sub-elite matches. This reveals important information for practitioners, as the physical training could be tailored to the game demands, and/or players could be moved between levels to assist in talent development. Future research should seek to improve our understanding of positional demands related to different tactics and team formations, and to investigate training load according to positional demands. More research is also necessary to better understand the influence of different contextual factors in youth soccer.

## 5. Conclusions

Physical performance during matches in this group of Norwegian U14 male soccer players was similar or higher than reports from other parts of the world and are highly related to playing position. Total distance and high-intensity running were greater in elite matches compared with sub-elite matches, but no differences were found regarding sprints, accelerations and decelerations. Match result and match arena also influenced running performance.

## Figures and Tables

**Table 1:** An overview of total match distance covered and high-intensity actions in Norwegian U14 players. Data are presented as means ± SD. Match playing time was 2 × 35 min. One-way ANOVA was used when comparing match halves independently of whether the same players played one or both halves.

	First Half (*n* = 187)	Second Half (*n* = 155)	Full Match	*p*-Values	ES Values
Total distance					
m	3883 ± 405	3762 ± 435	7645 ± 840	0.008	0.71
m·min^−1^	111.0 ± 11.6	107.5 ± 12.4	109.3 ± 12.0	0.008	0.29
HIR (13.6–18.5 km·h^−1^)					
m	618 ± 185	600 ± 199	1218 ± 398	0.365	0.09
m·min^−1^	17.7 ± 5.3	17.1 ± 5.7	17.4 ± 5.5	0.365	0.11
Sprint distance					
>18.5 km·h^−1^ (m) ^a^	242 ± 105	229 ± 116	471 ± 221	0.260	0.12
>25.2 km·h^−1^ (m) ^b^	19.8 ± 31.2	20.8 ± 31.1	40.6 ± 62.3	0.767	0.03
Maximal speed (km·h^−1^)	27.0 ± 2.2	26.6 ± 2.2	27.0 ± 2.2	0.061	0.18
Accelerations (*n*)	18.9 ± 9.4	19.7 ± 8.9	38.6 ± 18.3	0.419	0.09
Declarations (*n*)	23.1± 11.3	24.7 ± 10.9	47.8 ± 22.2	0.194	0.14

HIR: high-speed running. ^a^ threshold adapted from previous studies on U14 soccer players, ^b^ above 25.2 km·h^−1^ for more than 1 s (threshold adapted from studies on senior soccer players). Accelerations/decelerations: actions (>13.6 km·h^−1^) when speed was increased or decreased by more than 3 m·s^−2^ and lasted more than 0.5 s. Data were not corrected for multiple match observations by the same player. *p* ≤ 0.05 is statistically significant. ES: 0–0.2 = trivial; >0.2 = small; >0.5 = medium; >0.8 = large.

**Table 2:** Positional differences (mean ± 95% CI) in match running performance during one match half (35 min).

	Total Distance (m)	HIR (m) (13.6–18.5 km·h^−1^)	Sprints (m) (>18.5 km·h^−1^)	Accelerations (*n*)	Decelerations (*n*)
FB ‡	3987 [3838, 4136] (‡ > §, ∞) *	722 [653, 790] (‡ > §, ∞) *	281 [240, 322] (‡ > §, ¶) *	19.3 [16.3, 22.3]	25.2 [21.7, 28.6] (‡ > §) *
WM †	4015 [3883, 4148] († > §, ∞) *	712 [651, 774] († > §, ∞) *	288 [251, 326] († > §, ¶) *	20.0 [17.1, 22.9]	27.9 [24.5, 31.3] († > §, ¶) *
CB §	3673 [3506, 3839]	552 [475, 629]	187 [141, 233]	16.2 [13.1, 19.3]	18.0 [14.5, 21.5]
CM ¶	4020 [3887, 4153] (¶ > §, ∞) *	736 [674, 796] (¶ > §, ∞) *	217 [180, 254]	17.5 [14.9, 20.1]	23.6 [20.5, 26.6] (¶ > §) *
CF ∞	3734 [3551, 3917]	614 [529, 699]	257 [205, 309] (∞ > §) *	24.8 [20.1, 28.6] (∞ > ‡, †, §, ¶) *	27.6 [23.2, 32.0] (∞ > §) *

HIR: high-intensity running; FB: fullback; WM: wide midfielder; CB: centre back; CM: centre midfielder; CF: centre forward. Accelerations/decelerations: actions (>13.6 km·h^−1^) when speed was increased or decreased by more than 3 m·s^−2^ and lasted more than 0.5 s. Data were corrected for multiple match observations by the same player and all independent variables. Pairwise comparisons for playing position were performed with Bonferroni correction. * *p* ≤ 0.05.

**Table 3:** Match running performance (mean ± 95% CI) in one match half (35 min) in U14 players according to competitive level.

	Total Distance (m)	HIR (m) (13.6–18.5 km·h^−1^)	Sprints (m) (>18.5 km·h^−1^)	Accelerations (*n*)	Decelerations (*n*)
U14 elite matches	3898 [3779, 4016] *	671 [616, 727] *	247 [216, 279]	17.9 [15.1, 20.6]	22.9 [19.6, 26.2]
U14 sub-elite matches	3752 [3659, 3845]	596 [554, 639]	245 [222, 267]	20.4 [18.7, 22.2]	24.4 [22.2, 26.5]

U14 elite: national U14 elite level; U14 sub-elite: highest regional U14 level. HIR: high-intensity running. Accelerations/decelerations: actions (>13.6 km·h^−1)^ when speed was increased or decreased by more than 3 m·s^−2^ and lasted more than 0.5 s. Data were corrected for multiple match observations by the same player. Pairwise comparisons for playing position were performed with Bonferroni correction. * *p* ≤ 0.05.

## Data Availability

The data presented in this study are available on request from the corresponding author. The data are not publicly available due to ethical restrictions.

## References

[1] Vieira L.H.P., Carling C., Barbieri F.A., Aquino R., Santiago P.R.P. (2019). Match running performance in young soccer players: A systematic review. Sports Med..

[2] Waldron M., Murphy A. (2013). A comparison of physical abilities and match performance characteristics among elite and subelite under-14 soccer players. Pediatr. Exerc. Sci..

[3] Atan S.A., Foskett A., Ali A. (2016). Motion Analysis of Match Play in New Zealand U13 to U15 Age-Group Soccer Players. J. Strength Cond. Res..

[4] Arruda A.F., Carling C., Zanetti V., Aoki M.S., Coutts A.J., Moreira A. (2015). Effects of a very congested match schedule on body-load impacts, accelerations, and running measures in youth soccer players. Int. J. Sports Physiol. Perform..

[5] Al Haddad H., Simpson B.M., Buchheit M., Di Salvo V., Mendez-Villanueva A. (2015). Peak match speed and maximal sprinting speed in young soccer players: Effect of age and playing position. Int. J. Sports Physiol. Perform..

[6] Buchheit M., Mendez-Villanueva A., Simpson B.M., Bourdon P.C. (2010). Match running performance and fitness in youth soccer. Int. J. Sports Med..

[7] Mendez-Villanueva A., Buchheit M., Simpson B., Bourdon P.C. (2013). Match play intensity distribution in youth soccer. Int. J. Sports Med..

[8] Saward C., Morris J., Nevill M., Nevill A.M., Sunderland C. (2016). Longitudinal development of match-running performance in elite male youth soccer players. Scand. J. Med. Sci. Sports.

[9] Castellano J., Blanco-Villaseñor A., Alvarez D. (2011). Contextual variables and time-motion analysis in soccer. Int. J. Sports Med..

[10] Mohr M., Krustrup P., Bangsbo J. (2003). Match performance of high-standard soccer players with special reference to development of fatigue. J. Sports Sci..

[11] Nedelec M., McCall A., Carling C., Legall F., Berthoin S., Dupont G. (2014). The influence of soccer playing actions on the recovery kinetics after a soccer match. J. Strength Cond. Res..

[12] Grendstad H., Nilsen A.K., Rygh C.B., Hafstad A., Kristoffersen M., Iversen V.V., Nybakken T., Vestbostad M., Algroy E.A., Sandbakk O. (2019). Physical capacity, not skeletal maturity, distinguishes competitive levels in male Norwegian U14 soccer players. Scand. J. Med. Sci. Sports.

[13] Beato M., Coratella G., Stiff A., Iacono A.D. (2018). The Validity and Between-Unit Variability of GNSS Units (STATSports Apex 10 and 18 Hz) for Measuring Distance and Peak Speed in Team Sports. Front. Physiol..

[14] Beato M., de Keijzer K.L. (2019). The inter-unit and inter-model reliability of GNSS STATSports Apex and Viper units in measuring peak speed over 5, 10, 15, 20 and 30 meters. Biol. Sport.

[15] Castagna C., D’Ottavio S., Abt G. (2003). Activity profile of young soccer players during actual match play. J. Strength Cond. Res..

[16] Ingebrigtsen J., Dalen T., Hjelde G.H., Drust B., Wisløff U. (2015). Acceleration and sprint profiles of a professional elite football team in match play. Eur. J. Sport Sci..

[17] Bradley P.S., Sheldon W., Wooster B., Olsen P., Boanas P., Krustrup P. (2009). High-intensity running in English FA Premier League soccer matches. J. Sports Sci..

[18] Cohen J. (1988). Statistical Power Analysis for the Behavioral Sciences.

[19] Harley J.A., Barnes C.A., Portas M., Lovell R., Barrett S., Paul D., Weston M. (2010). Motion analysis of match-play in elite U12 to U16 age-group soccer players. J. Sports Sci..

[20] Castagna C., Impellizzeri F., Cecchini E., Rampinini E., Alvarez J.C. (2009). Effects of intermittent-endurance fitness on match performance in young male soccer players. J. Strength Cond. Res..

[21] Yi Q., Groom R., Dai C., Liu H., Gómez Ruano M.Á. (2019). Differences in Technical Performance of Players From ‘The Big Five’ European Football Leagues in the UEFA Champions League. Front. Psychol..

[22] Sarmento H., Pereira A., Matos N., Campaniço J., Anguera T.M., Leitão J. (2013). English Premier League, Spaińs La Liga and Italýs Seriés A–What’s Different?. Int. J. Perform. Anal. Sport.

[23] Dellal A., Chamari K., Wong D.P., Ahmaidi S., Keller D., Barros R., Bisciotti G.N., Carling C. (2011). Comparison of physical and technical performance in European soccer match-play: FA Premier League and La Liga. Eur. J. Sport Sci..

[24] Vigh-Larsen J.F., Dalgas U., Andersen T.B. (2018). Position-Specific Acceleration and Deceleration Profiles in Elite Youth and Senior Soccer Players. J. Strength Cond. Res..

[25] Buchheit M., Simpson B.M., Mendez-Villanueva A. (2013). Repeated high-speed activities during youth soccer games in relation to changes in maximal sprinting and aerobic speeds. Int. J. Sports Med..

[26] Bradley P.S., Carling C., Archer D., Roberts J., Dodds A., Di Mascio M., Paul D., Diaz A.G., Peart D., Krustrup P. (2011). The effect of playing formation on high-intensity running and technical profiles in English FA Premier League soccer matches. J. Sports Sci..

[27] Pettersen S.A., Brenn T. (2019). Activity Profiles by Position in Youth Elite Soccer Players in Official Matches. Sports Med. Int. Open.

[28] Bush M., Barnes C., Archer D.T., Hogg B., Bradley P.S. (2015). Evolution of match performance parameters for various playing positions in the English Premier League. Hum. Mov. Sci..

[29] Varley M.C., Gregson W., McMillan K., Bonanno D., Stafford K., Modonutti M., Di Salvo V. (2017). Physical and technical performance of elite youth soccer players during international tournaments: Influence of playing position and team success and opponent quality. Sci. Med. Footb..

[30] Di Salvo V., Gregson W., Atkinson G., Tordoff P., Drust B. (2009). Analysis of high intensity activity in Premier League soccer. Int. J. Sports Med..

[31] Lago C., Casais L., Dominguez E., Sampaio J. (2010). The effects of situational variables on distance covered at various speeds in elite soccer. Eur. J. Sport Sci..

[32] Lago C. (2009). The influence of match location, quality of opposition, and match status on possession strategies in professional association football. J. Sports Sci..

[33] Bradley P.S., Lago-Penas C., Rey E., Sampaio J. (2014). The influence of situational variables on ball possession in the English Premier League. J. Sports Sci..

[34] De Hoyo M., Cohen D.D., Sañudo B., Carrasco L., Álvarez-Mesa A., Del Ojo J.J., Domínguez-Cobo S., Mañas V., Otero-Esquina C. (2016). Influence of football match time–motion parameters on recovery time course of muscle damage and jump ability. J. Sports Sci..

